# Interlink between the gut microbiota and inflammation in the context of oxidative stress in Alzheimer’s disease progression

**DOI:** 10.1080/19490976.2023.2206504

**Published:** 2023-05-01

**Authors:** Tushar K. Das, Bhanu P. Ganesh

**Affiliations:** Department of Neurology, McGovern Medical School, University of Texas Health Science Center at Houston, Houston, TX, USA

**Keywords:** Microbiota-gut-brain axis, gut microbiota, dysbiosis, oxidative stress, inflammation, Alzheimer’s disease

## Abstract

The microbiota-gut-brain axis is an important pathway of communication and may dynamically contribute to Alzheimer’s disease (AD) pathogenesis. Pathological commensal gut microbiota alterations, termed as dysbiosis, can influence intestinal permeability and break the blood–brain barrier which may trigger AD pathogenesis via redox signaling, neuronal, immune, and metabolic pathways. Dysbiosis increases the oxidative stress. Oxidants affect the innate immune system through recognizing microbial-derived pathogens by Toll-like receptors and initiating the inflammatory process. Most of the gut microbiome research work highlights the relationship between the gut microbiota and AD, but the contributory connection between precise bacteria and brain dysfunction in AD pathology cannot be fully demonstrated. Here, we summarize the current information of the fundamental connections between oxidative stress, inflammation, and gut dysbiosis in AD. This review emphasizes on the involvement of gut microbiota in the regulation of oxidative stress, inflammation, immune responses including central and peripheral cross-talk. It provides insights for novel preventative and therapeutic approaches in AD.

## Introduction

Alzheimer’s disease (AD) is the most common cause of neurodegenerative disorders such as dementia. Cerebral extracellular amyloid β (Aβ) aggregation and intracellular neurofibrillary tangles (NFTs) formation are the primary histological hallmarks of AD. ^1^ Now a days, AD is the foremost global health affliction worldwide. The global prevalence of dementia rises exponentially with age. At 60–64 years it is around 0.7–1.8%, and over 90 years it goes up to 63.9% worldwide.^2^ In 2021, about 6.2 million Americans were affected by AD; this number is projected to rise to nearly 13.8 million by 2060. Although the death rate by stroke, heart disease, and HIV were reduced between 2000 and 2019; unfortunately, AD death rate was increased by around 145%. During COVID-19 pandemic, AD death rate was increased by 6% in the USA.^3^ In 2021, the total cost of healthcare-related dementia was estimated at around $355 billion in the USA.^3^ Though the precise biological changes of AD, the different rates of progression among affected individuals and how AD can be prevented, slowed down, or stopped are still largely mysterious. Therefore, studying the mechanism of AD pathogenesis and finding new treatment strategies for the prevention and cure of AD is one of the most important challenges to be tackled in AD research.

Numerous evidences support that oxidative stress is one of the important cause of cell damage in AD^4,5^. The abundance of oxidative products which alter the major histopathology are increased in the AD brain.^6^ Oxidative stress is mostly generated by reactive oxygen species (ROS) and reactive nitrogen species (RNS). Extreme accumulation of ROS induces neuronal damage.^4–6^ Clearing or inhibiting the surplus ROS/RNS from the brain may be a fruitful treatment of AD. Oxidative stress will be discussed more in detail in the sections below.

**Figure 1.**
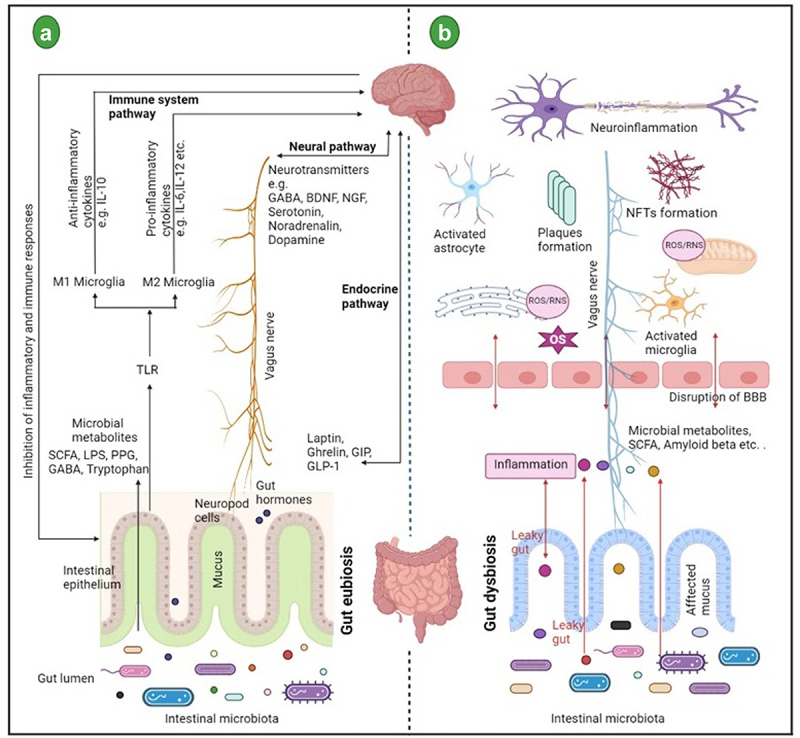
(a) Schematic representation of bidirectional communication between gut and brain through “microbiota-gut-brain axis”. The communications are mainly carried out by neural, endocrine, and immunological pathways. (b) in the lumen gut microbiota, microbial-derived metabolites such as short-chain fatty acids (SCFAs), neurotransmitters, amino acids, and bacterial amyloid interacts with the host immune system. These interactions affect the host metabolism and may activate the vagus nerve. Therefore these interactions are key to maintaining the overall health of the host. During dysbiosis, unfavorable conditions may cause the activation of corticotropin receptor, subsequently triggering adrenocorticotrophic hormone release and finally influencing cortisol release which leads to the loss of intestinal barrier integrity.^9,10^ As a result, intestinal and blood-brain barrier permeability is increased. Due to increased permeability, there is an increase in reactive oxygen species (ROS) in the neurons and microglia which may cause oxidative stress in neurodegenerative diseases such as Alzheimer’s disease.

Recent evidence suggested that age-related attenuation of gut microbiota biodiversity is considered as an important reason of AD pathogenesis.^7,8^ Gut microbiota can regulate the multiple neurochemical pathways through the “microbiota-gut-brain axis”. “microbiota-gut-brain axis” states to a bidirectional network communication between the central nervous system (CNS) and the gastrointestinal (GI) tract connecting various overlying pathways such as the autonomic, neuroendocrine, and immune systems including bacterial metabolites and neuromodulator molecules directly affecting the brain function (Figure 1). Although the microbiota-gut-brain axis helps the appropriate function of the digestive tract, it also controls the biochemical signals between the sympathetic nervous system, endocrine glands, and specific brain regions such as the hypothalamus and the frontal cortex.^11^

Gut microbiota communicates to the brain through four significant routes; first, vagus nerve activation which joins the muscular and mucosal layer of the GI tract to the brain stem; secondly, secretion of serotonin from enterochromaffin cells (EC) which are present in the gut epithelial lining; thirdly, dysfunction of microglia; and last direct transfer of the chemical signals (toxins, short-chain fatty acids, γ-aminobutyric acid, etc.) to the brain.^12–14^ These four routes may work in combination to progress Aβ signaling cascade from the gut to the brain.

GI tract encloses trillions of commensal microorganisms and~1000 of its species which regulate a variety of metabolic functions and preserve membrane barrier functions mainly in the gut.^9^ Within two years after birth, the community of microbes stabilizes the host GI tract, but depending upon peripheral factors such as age, diet, health, genetics, lifestyle, and environment differ their composition among individuals.^10^ Furthermore, neurotransmitter γ-aminobutyric acid (GABA) can be produced by some beneficial gut microbiota like *Bacteroides, Bifidobacterium*, *Parabacteroides*, and *Escherichia spp*. Therefore, gut microbiota controls the level of neurotransmitters in the host organism.^15^ With increasing age, pathological changes in the gut microbiota composition, termed as dysbiosis leads to inflammation, disrupts the blood–brain barrier (BBB), activates the immune system, and produces ROS and this is also seen in AD circumstances^16^(Figure 1). Increasing abundance of *Escherichia, Shigella spp., Bacteriodetes*, and decreasing population of *Bifidobacterium spp*. with disturbed bacteriodetes versus firmicutes ratio might quicken inflammation and Aβ aggregation in AD.^17,18^ Therefore, a healthy well-balanced gut microbiome may reduce or prevent the detrimental effects of oxidative stress in AD. Here, we summarize the current research work on the association between oxidative stress and the gut microbiota in AD. In a nutshell, this review will provide evidence of the interlink between the gut microbiota and inflammation in the context of oxidative stress and the role of gut-microbial metabolites in AD.

## Oxidative stress in AD

Oxidative stress is known as the imbalance between the ROS/RNS and antioxidant levels in cells. It disrupts the redox signaling pathway and contributes to microglial dysfunction in AD^19^. ROS are mainly formed as the secondary product of the leaky electron transport chain (ETC) (complex I and III) in mitochondria.^4^ In addition, monoamine oxidases (MAO) (present in the outer mitochondrial membrane),^20^ an isoform of nitric oxide synthase (NOS) (present in neurons),^21^ NADPH oxidases (NOX) (present in the plasma membrane, and phagosomes of polymorphonuclear neutrophils, abundance in cortex and hippocampus regions of the brain),^22^ non-heme iron enzymes such as lipoxygenases (present in the cytoplasm),^23^ xanthine oxidase, cytochrome P450 monooxygenase, cyclooxygenase and D-amino oxidase (present in the cytoplasm)^24^ are important ROS producers. Peroxisomes also have a contribution to ROS formation.^25^ Oxidative stress is augmented by the formation of superoxide (O_2_.−) by one electron from the molecular oxygen (O_2_), hydrogen peroxide (H_2_O_2_), peroxynitrite (ONOO−), and hydroxyl radicals (.OH) which are produced by the Fenton and Haber Weiss reaction in AD. The multi-valence of transition metals such as iron, copper, aluminum, and zinc help free radical formation causing oxidative stress. Lipid peroxidation, protein oxidation, nucleic acid damage, and advanced glycation end-products (AGEs) formation are the main four reactions which cause cellular damage in oxidative stress.^4^ Oxidative stress biomarkers such as malondialdehyde, 4-hydroxynonenal, and F2-isoprostane (lipid oxidative damage); protein carbonyls and 3 nitrotyrosine (products of protein oxidation), 8-hydroxydeoxyguanosine (nucleic acid oxidation) are observed at high concentration in the blood and the cerebrospinal fluid (CSF) of AD patients.^26^

**Figure 2.**
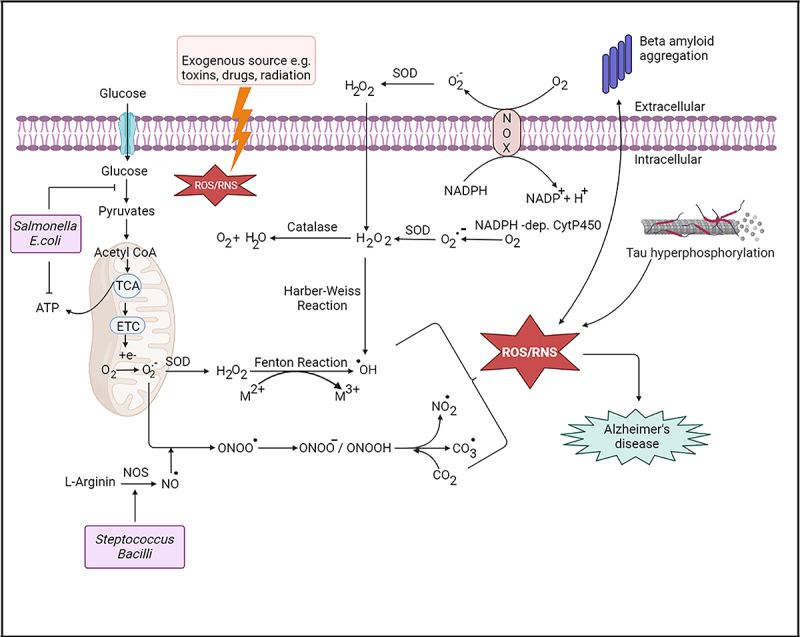
Schematic representation of reactive oxygen species (ROS) and reactive nitrogen species (RNS) formation in AD brain. Mitochondria is the main ROS producer. ROS are produced through electron leak from aerobic respiration in mitochondria. Superoxide (O_2_.−) is converted to hydrogen peroxide (H_2_O_2_) by superoxide dismutases (SOD). O_2_.− is also formed by the oxidation of NADPH by NADH oxidase enzymes (NOXs). Oxygen is produced by the reaction with H_2_O_2_ and catalase. In addition, H_2_O_2_ can produced hydroxyl radical (OH.) by Fenton and Haber–Weiss reaction in the presence of metal ions. On the other hand, reactive nitrogen species such as NO., CO_3_. are produced by the reaction of peroxide ions (ONOO.) and (O_2_.−). Exogenous source such as toxins or radiation may cause ROS/RNS production. ROS production may accelerate amyloid beta (Aβ) aggregation and tau hyperphosphorylation in Alzheimer’s disease., Streptococcus and Bacilli increases RNS production; whereas Salmonella and E. coli inhibit ATP production which accelerates oxidative stress, inflammation and thus may significantly influence amyloid-beta aggregation in AD.

In swift, dysfunction in cellular organelles like mitochondria^27^, endoplasmic reticulum due to unfolded protein response (UPR)^28^, augmentation of metal ions in neuritic plaques^29^ and hyperactivation of microglia followed by the upregulation of NADPH oxidase^30^ are characterized by ROS/RNS production^31^ in AD. Oxidative stress can accelerate the aggregation of Aβ and vice versa.^4,26^ Additionally, hyperphosphorylated tau proteins may lead to reduced activity of NADH-ubiquinone reductase enzyme, increasing ROS production and mitochondrial dysfunction in AD.^32^ Therefore, the relationship between oxidative stress and AD (Figure 2) is well documented in previous studies which indicate the importance of the antioxidant defense system in the brain.

### Oxidative stress: the role of gut microbiota

By the modulation of mitochondrial activity, commensal and pathogenic bacteria can change the cellular oxidative stress in the gut^33^. Formylated peptides are produced by commensal bacteria and it binds with G protein-coupled receptors (GPCRs) on macrophages and neutrophils and trigger inflammation^34,35^. As a result, superoxide is produced by NOX-1 which increases cellular ROS.^36^ In addition, nitrate and nitrites compounds can be converted to nitric oxide (NO) by the gut *Lactobacilli* and *Bifidobacterium*, creating a high abundance of NO in the gut epithelia. NO is also produced from L-arginine using NOS by *Streptococcus* and *Bacilli*. ^37^ Although the nanomolar concentration of NO is considered as a neuroprotective and neurotransmitter of noradrenergic, noncholinergic enteric neurons; the higher concentration of NO produces ROS/RNS which further forms hydroxyl radicals, resulting in detrimental effects to neuroinflammation, axonal degeneration, and neurodegenerative disorders.^38^ Furthermore, *Salmonella typhimurium, E. coli, Mycobacterium*, and *Streptococcus anginosus* can produce hydrogen sulfide from sulfur-containing amino acids (e.g. Cysteine) in the GI tract by sulfur metabolism. Due to the high concentration of hydrogen sulfide, cyclooxygenase activity is inhibited which alters the metabolism toward glycolysis, resulting in the reduction of mitochondrial oxygen consumption, ATP production, and overexpression of pro‐inflammatory effects (Figure 2).^39,40^ Additionally, dietary trimethylamine N‐oxide (TMAO) is accelerated oxidative stress by reducing superoxide dismutase levels, increasing malondialdehyde and glutathione peroxidase, and exacerbating inflammation by the production of proinflammatory cytokines such as IL-6, IL-10, IL-1β, TNF-∞ in plasma and liver of male Apolipoprotein E knock-out (ApoE−/−) mice.^41^

## Oxidative stress and inflammation in AD

Oxidative stress and inflammation are intimately connected in the pathophysiological conditions where redox homeostasis is interrupted.^42^ Growing evidences indicate that inflammation/neuroinflammation is a significant contributor to AD development and exacerbation.^42,43^ Inflammation is mainly arbitrated by microglial and astroglial states in the brain. Microglia have an important role in the brain development and function. Deposition of Aβ increases neuronal injury and inflammation by triggering microglia activation in AD. Activated microglia release some pro-inflammatory cytokines such as IL-6, TNF-∞ and IL-1β which regulates the inflammation.^44^ Interestingly, IL-1β regulates the amyloid precursor protein, parent protein of Aβ.^45^ The CSF- IL-1β is higher in AD than in age-matched control patients.^46^ By the activation of p38 mitogen-activated protein kinase and glycogen synthase kinase-3β pathway, highly concentrated IL-1β accelerates tau hyperphosphorylation and NFT formation in triple transgenic AD mouse model.^47^

Additionally, microglia have a significant role in Aβ clearance and degradation.^48^ During microglial phagocytosis over time, the efficiency of microglia gradually declines Aβ clearance, which reduces Aβ proteolysis^49^ and increases Aβ deposition and aggregation^50^. This incidence creates an overproduction of pro-inflammatory cytokines, which trigger more ROS production and AD onset^19^. Therefore, microglia is one of the important non-neuronal cells for plaque formation and oxidative stress initiation in the AD brain.

Microglial degeneration can be occurred by stimulating receptor expressed on myeloid cells 2 (TREM2), C×3C motif chemokine receptor 1 (CX3CR1), GABA, and other inflammatory cytokine mediators. The expression of TREM2 on the microglial cell surface increases the phagocytic activity and causes ROS production.^51^ During the phagocytosis activity microglia produce ROS like O_2_.. Active microglia also generate O_2_^−.^ via NOX-2 pathway. H_2_O_2_ and NO. also impose local inflammation by attracting microglia which are involved in oxidative stress^19^ that may drive AD progression.

Accumulating evidences showed that the astrocytes are intensively involved in maintaining oxidative stress at physiological or pathological conditions.^52,53^ Astrocytes are playing a dual role in maintaining homeostasis of ROS/RNS regulation.^52^ Under physiological conditions, astrocyte acts as a neuroprotector of CNS from oxidative injury. In this condition various antioxidants are secreted, endogenic antioxidative systems like nuclear factor E2-related factor 2 (Nrf2) is stimulated, the excitatory amino acids are removed, neurotransmitters are uptaken and metabolized, energy and neurotrophin are produced and finally ROS/RNS are degraded.^52,54^ Whereas, under pathophysiological conditions, astrocytes trigger by stimulation from microglial activation and neuronal degradation. As a result, excessive ROS/RNS are produced from impaired mitochondria and antioxidant production is reduced; and elevated pro-inflammatory cytokines which leads to a detrimental effect seen in AD. Aβ and pro-inflammatory cytokines can trigger the astrocytes, causing instigation of NF-kB pathway, subsequently more pro-inflammatory cytokines and chemokines are produced .^55^ Astrocytes are the vital cells that control glutamate homeostasis which circuitously maintains oxidative stress.^56^ In the pathological circumstance, excessive glutamate is secreted from the pre-synaptic membrane and collected in the synaptic cleft which may allow a large influx of Ca^2+^ by over-activating N-methyl-d-aspartate (NMDA) and α-amino-3-hydroxy-5-methyl-4-isoxazolepropionic acid (AMPA) receptors. It starts with calcium overload and ROS production in mitochondria; and causes neurotoxicity.^57^

Oligodendrocyte, the myelin-forming cells (myelinate neuronal axons) contribute to increased oxidative stress in the CNS. Oligodendrocytes have lower glutathione content and it increases the iron content in the brain, which makes them further susceptible to oxidative stress and damage.^58^ Oligodendrocytes are also exposed to Aβ and lead to oxidative stress, resulting in demyelination.^59^ Demyleination causes a reduction in neuronal action potential time with increased inflammation and oxidative stress, thereby contributing to cognitive impairment in AD.

### Oxidative stress and inflammation: role of the gut microbiota

Gut microbiota plays numerous roles in the improvement of immune response, protection against pathogen colonization^60^ at the intestinal epithelial barrier^61^, association with production/regulation of oxidative stress and inflammation^62^ in the host. Preclinical^63^ and clinical studies^64,65^ show that modifications in gut microbiota is connected with AD progression. Gram-negative bacteria such as *E. coli* and *Shigella* colonization leads to surge in the formation of bacterial amyloids and lipopolysaccharides (LPS) which causes peripheral systemic inflammation, resulting in dysfunctional GI permeability and impaired BBB function.^66,67^ Dysbiosis causes increase in pathogenic bacteria such as *Escherichia*, *Shigella*, *Pseudomonas*, *Proteobacteria, and Verrucomicrobia* and reduces beneficial bacteria e.g. *Bifidobacterium*, *Bacteroides fragilis, Bacillus fragilis, Eubacterium hallii, Eubacterium rectale*, and *Faecalibacterium prausnitzii*.^18^ Gut microbial metabolites like indole-3-pyruvic acid and short-chain fatty acids (SCFAs) were gradually decreased from mild cognitive impairment (MCI) to AD patients.^68^ The abundance of SCFA-producing bacteria such as *Clostridia*, *Clostridiales Ruminococcaceae*, *Firmicutes*, and *Ruminococcus* declined which indicates the AD progression by the host-microbe cross-talk signals.^68^ Other studies revealed that *Blautia*, *Desulfovibrio*, *Escherichia*‐*Shigella*, and *Akkermansia* are markedly altered in the APP/PSI transgenic mice.^69,70^ High abundance of Enterotype I and III bacteria are connected with the incidence of dementia.^71^ The level of *Bifidobacterium*, *Blautia*, *Lactobacillus*, and *Sphingomonas* were more than *Anaerobacterium*, *Papillibacter*, and *Odoribacter* in AD patients.^72^ Additionally, the affluence of *Firmicutes*, *Proteobacteria, Tenericutes Enterobacteriaceae, Coriobacteriaceae, Mogibacteriaceae, Phascolarcto-bacterium*, and *Coprococcus* were higher in MCI patients than age-matched controls.^73^ The abundances of *Firmicutes, Actinobacteria, E. rectale, B. fragilis* and *Fusobacteriaceae* were decreased whereas, the levels of *Bacteroides, Tenericutes, E. coli, B. subtilis, Escherichia, shigella, Lactobacilli, Bifidobacteria, Prevotellaceae, Verrucomicrobia* were increased in the feces when compared with respective control groups in both AD animal models and AD patients^74^. The higher abundance of fungi like, *Phaffomyceteceae, Sclerotiniaceae, Cystofilobasidiace-ae, Togniniaceae, Trichocomaceae*, *Botrytis, Cladosporium, Kazachstania, Phaeoacremonium* and lower affluence of *Meyerozyma* were found in MCI patients.^75^ For example, dietary amines can be metabolized to trimethylamine by gut microbiota whereas liver upon nutrient absorption converts trimethylamine to TMAO. Trimethylamine availability can be influenced by the gut dysbiosis. Increased levels of TMAO can promote the upregulation of inflammatory cytokines that raise oxidative stress in AD.^76^ TMAO were found to be elevated in the cerebrospinal fluid of AD and MCI patients.^77^

Therefore, beneficially modifying the gut microbiota content can show an optimistic role in the declining ROS through SCFA such as butyrate production, whereas dysbiosis may accelerate the systematic inflammation, activation of microglia, and BBB damage by elevated TMAO and further promotes AD progression.^78^

### Interlink between oxidative stress, inflammation, and TLRs in AD

Toll-like receptors (TLRs) are type 1 transmembrane pattern recognition receptors that are composed of an extracytoplasmic leucine-rich repeat (LRR) domain, a single membrane-spanning helix, and a signaling Toll-interleukin-1 receptor (TIR) domain. Based on spatial distribution of TLRs, it is mainly divided into two groups. One group (TLR1, TLR2, TLR4, TLR5, TLR 6, and TLR11) are found on the plasma membranes and they can be activated by microbial products e.g. lipids, lipoproteins, and proteins. Other group (TLR3, TLR7, TLR8, and TLR9) is located in cytoplasmic compartments. It can be triggered by nucleic acid species.^79^

**Figure 3.**
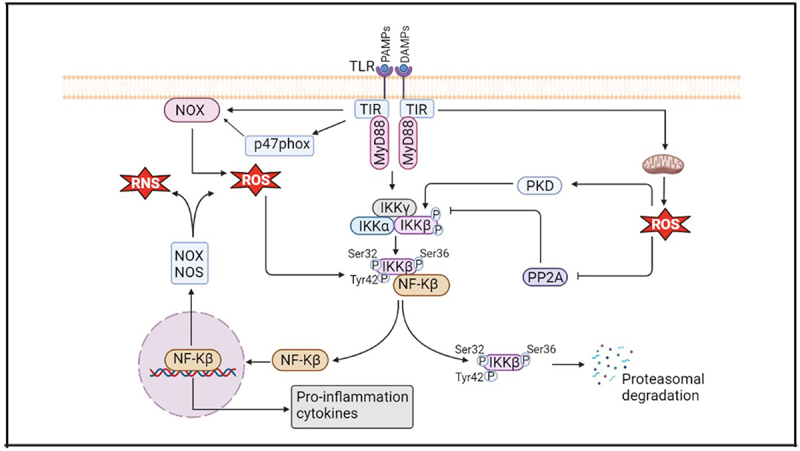
Schematic representation of TLR-mediated oxidative stress and inflammation in AD. TLRs help the formation of ROS through either direct interaction at the cell membrane or enhanced phosphorylation of its p47phox subunit within the cytoplasm. In the MyD88-dependent pathway, TLRs activate downstream signaling through the phosphorylation of IkB at Ser 32 and 36, and; it causes proteasomal degradation. Free nuclear factor kappa B (NF-Kb)) translocates to the nucleus and produces proinflammatory cytokines and oxidant enzymes which increases ROS levels. In addition, ROS accelerates the activation of NF-Kb by enhancement of IκB kinase (IKK) phosphorylation by activating PKD or inhibiting protein phosphatase type 2A (PP2A).

TLRs stimulate the downstream signaling transduction by identifying microbes-derived pathogens through the damage- and pathogen-associated molecular patterns (DAMPs and PAMPs) and start the inflammation. Firstly, TLRs change their confirmation based on PAMPs or DAMPs, and then dimerize to enhance the downstream signaling adaptors such as myeloid differentiation primary response protein 88 (MyD88), TIR domain-containing adaptor molecule (TIRAP), TIR domain-containing adaptor protein inducing interferon-b (TRIF) and TRIF-related adaptor molecule (TRAM) which stimulate the precise transcription factors and innate immune responses.^80^ Except for TLR3, MyD88 is a common adaptor protein for all TLR-mediated signaling pathways.^81^ Upon TLR involvement, the MyD88-dependent pathway can activate downstream signaling through the phosphorylation of IkB at ser 32 and 36 and it causes proteasomal degradation. As a result, free nuclear factor kappa B (NF-kb) translocates to the nucleus and activates targeted genes which produce proinflammatory cytokines such as TNF-α, IL-1, and IL-6 and oxidant enzymes such as NOX and iNOS which increases ROS levels. ROS accelerates NF-kB activation by enhancement of IKK phosphorylation by initiating protein kinase D (PKD) or obstructing protein phosphatase type 2A (PP2A).^82^ In addition, TLRs also help ROS production within mitochondria and activate NOX by straight communication at the cell membrane or by increasing the phosphorylation of its p47phox subunit within the cytoplasm.^82^ As a result, intracellular ROS level is increased and it helps to mobilize and dimerize TLRs and amplifies TLR responses (Figure 3). Therefore, TLR-mediated inflammation plays a significant role in oxidative stress.

Although 11 human and 13 mouse TLRs are identified, recent evidence indicates TLR2, TLR4, and TLR9 are mainly involved in AD onset (Table 1).^80–82,101^ Although, the function of TLR2 in AD is controversial, some researchers believe that TLR2 can identify Aβ42 and trigger pro-inflammatory cytokines (e.g. TNF-α, IL-6, and IL-1) secretion.^83,84^ It was found that decreased levels of Aβ and higher toxicity of Aβ 1–42 can cause more cognitive decline in TLR2 knockout APP/PS1 mice.^85^ It has been previously documented that uptake of Aβ42 by microglia is significantly increased by the activation of TLR2 expression.^86^

**Table 1.** The effects of Toll-like receptors in Alzheimer’s disease.

Type of Toll-like receptors	Research Models	Findings	Ref
TLR2	TLR2−/− mice	Triggered TNF-α, IL-6, and IL-1 secretion	^83^
TLR2	Mouse NG108–15 neural cell	Increased TNF-α, MCP-1 and IL-10 by TLR2/JNK/NF-κB pathway	^84^
TLR2	APP-TLR2(-/-) mice	Aβ stimulates TLR2 expression	^85^
TLR2	Mouse SDF-1 (stromal cell-derived factor 1)	Enhanced mFPR2-mediated uptake of Aβ42 by microglia	^86^
TLR2	Tg2576 mice	Bacterial amyloid activated TLR2 before Aβ pathology	^87^
TLR2	APP TgCRND8 transgenic mice	Upregulation of TLR2 and CD14	^88^
TLR2	APPswe/PS1dE9 mice	Triggered neuroinflammatory activation	^89^
TLR2	APP/PS1 mice	Developed inflammation and Aβ accumulation	^90^
TLR2	TLR2-deficient mice	Aβ increased TLR2 expression	^91^
TLR4	C3H/HeJ mice	Enhanced IL-6, TNFα and nitric oxide production	^92^
TLR4	Homozygous TLR4 mutant Mo/Hu APPswe PS1dE9 transgenic mice	Upregulated TNF-α, IL-1β, IL-10 and IL-17	^93^
TLR4	TgAPPswe/PS1dE9 mice	Increased Aβ deposition and soluble Aβ42 in the brain, expression levels of IL-1β, CCL3, and CCL4	^94^
TLR4	P301S-transgenic mice	Promoted inflammation and attenuated AD-related tauopathy	^95^
TLR4	TgAPP/PS1 mice	Triggered Aβ accumulation in brain	^96^
TLR9	Mouse TLR9-myc cDNA	Decreased mitochondrial ATP levels	^97^
TLR9	Sporadic late-onset AD Patient	Decreased late-onset AD risk	^98^
TLR9	Tg-SwDI mice	Stimulated the innate immune system and microglia/macrophage activation	^99^
TLR9	3xTg-AD mice	Ameliorated microglial function	^100^

Recently, we found that the integrity of the gut barrier was markedly decreased in Tg2576 AD mice which might increase bacterial amyloid (curli) burden in the gut before the appearance of Aβ deposition in the brain.^87^ Curli stimulated TLR2 activation and co-localized with neuroendocrine marker PGP 9.5 within the epithelium and sub-mucosa of the gut in AD mice.^87^ It indicated the vagus nerve activation by bacterial curli. Therefore, activation of TLR2 can stimulate AD pathogenesis^87,88,92,102^ It indicates that inhibition of TLR2 from periphery might be beneficial for AD. *In-vivo* and *in-vitro* studies also showed that phagocytosis and Aβ clearance in microglia were increased by the deficiency of TLR2^89^. Secretion of pro-inflammatory cytokines and Aβ accumulation was reduced by the inhibition of TLR2 which improved the spatial learning performance in AD mouse models.^90,91^

TLR4 also plays a double role in AD pathogenesis. Jin et al. found that some cytokines such as IL-1β, IL-10, IL-17, and TNF-α were increased in TLR4 mutant Mo/Hu APPswe PS1dE9 transgenic mice (TLR4M AD mice), indicating TLR4 signaling may be involved in AD pathogenesis.^93^ Song et al. also observed that TLR4 mutation decreased the microglial activation and accelerated Aβ deposition in AD mice, suggesting that microglial TLR4-mediated Aβ-induced neurotoxicity increases the clearance of Aβ deposition in the brain.^94^ In addition, Qin et al. found that neuroinflammation could help neuronal autophagy, indicating mild TLR4 stimulation that attenuates AD-related tauopathy in tau-transgenic AD mice.^95^ Go et al. found that TLR4 signaling in microglia was changed in the AD mouse model (TgAPP/PS1) and suggested the alteration of TLR4 signaling might help understand Aβ accumulation in the brain.^96^

In addition, recent evidence indicated that activation of TLR9 signaling can defend neurons from stress;^97^ Polymorphism of TLR9 may reduce the risk of AD^98^ and TLR9 knockout mice showed impaired synaptic function.^99^ Use of TLR9 agonists in AD mice cause the levels of Aβ aggregation and tau hyperphosphorylation declined which might improve cognitive deficits.^100^ Therefore, immunomodulation through TLR9 may act as a probable therapeutic approach for AD but needs further investigation.

## Oxidative stress and Nrf2-Keap 1 pathway in AD

**Figure 4.**
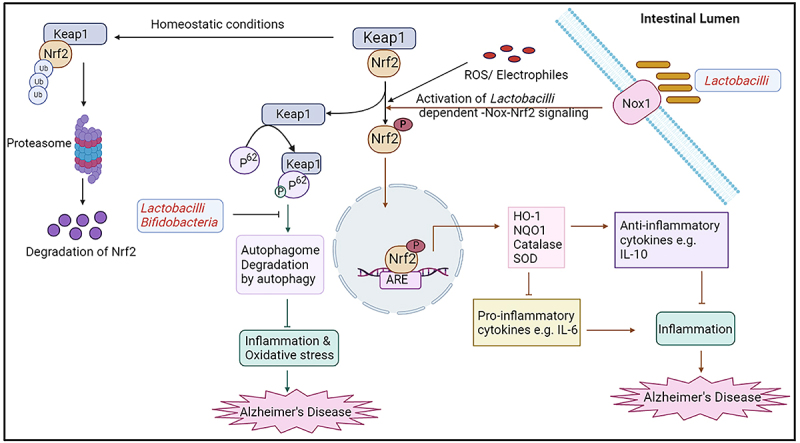
Schematic representation of the role of gut microbiota in Nrf2-keap 1 pathway and autophagy in AD. In homeostatic conditions, Nrf2 is degraded through proteasomal degradation. In oxidative stress conditions, Nrf2 dissociates from Keap1 and Nrf2 transport into the nucleus, binds with antioxidant response elements (ARE), and finally produces HO-1, NQo1, Catalase, and SOD. As a result, levels of anti-inflammatory cytokines are increased and pro-inflammatory cytokines levels are decreased. Lactobacilli can prompt their beneficial influence on host gut-epithelial tissue by the activation of lactobacilli-Nox-Nrf2 signaling. Lactobacilli and Bifidobacteria might help to decrease p62 levels and increase beclin-1 and LC3-II by activation of autophagic flux. Therefore, increased levels of Nrf2 and decreased levels of p62 can act as a neuroprotectant in AD.

Cellular central defense mechanism against oxidative stress is regulated by the nuclear factor E2-related factor 2 (Nrf2)-Kelch-like ECH-associated protein 1 (Keap1) signaling pathway^103–105^. Under homeostatic condition with low abundance of ROS, Keap1 binds with Nrf2 and then cullin-dependent E3 ubiquitin ligases the Nrf2 by proteasomal degradation. Under stimulation of elevated ROS levels, Nrf2 detaches from Keap1 and then Nrf2 transports into the nucleus, binds with antioxidant response elements (ARE) and finally promotes to increase downstream antioxidant enzyme genes (GCLC, GCLM, HO-1, and NQO1) which reduces the oxidative stress in AD^106^. Numerous evidences reported that Nrf2 expression level are progressively reduced in the brain with the increasing of age. That leads to poorer clearance of resulting ROS immediately from the cytoplasm with age^107–109^. Consequently, oxidative damage and synaptic structural damage occurs in the neuronal cell, which is one of the primary reasons for AD. From current accumulating evidences, it is indicated that the activation of Nrf2-Keap 1 pathway ameliorates oxidative stress in AD (Figure 4).

### Oxidative stress and Nrf2-Keap 1 pathway: role of the gut microbiota

Numerous evidences suggest that cellular ROS is produced in the gut epithelial cells by the catalytic action of NADPH oxidases to the gut bacteria.^110,111^ Enzymatically ROS production by NADPH oxidase 1 (Nox1)^112^ in epithelia is accelerated by pathogens, whereas symbiotic bacteria like *Lactobacilli* controls the intestinal epithelial cell proliferation^113^, recompensation post-injury^114^, and modification of epithelial NF-kB signaling.^115^ Jones et al. found that Nox1 is required to activate the Nrf2 pathway for the epithelial cytoprotection which is mediated by the gut bacteria *Lactobacilli* in the Drosophila and mice. Activated Nrf2 pathway boosts cytoprotective genes against environmental oxidative stress (Figure 4). Therefore, *Lactobacilli* prompts their promising influence on host gut-epithelial tissue by the activation of lactobacilli dependent-Nox-Nrf2 signaling.^116^ Therefore, Nrf2 signaling pathway initiates cytoprotection within the gut epithelial cells.^117^

### Crosstalk between autophagy and Nrf2 pathway in AD

Autophagy is a significant metabolic process which eliminates the misfolded proteins in the cells. Inception of autophagy and autophagosomes formation is other promising pathway for the treatment of AD.^107^ Initiation of the Nrf2 pathway and autophagy are both helpful for the reduction of AD progression. There is a reciprocal relationship between autophagy and oxidative stress. After the elimination of Keap1 from Nrf2, keap1 binds with phosphorylated ubiquitin-binding protein p62 and finally triggers the autophagy by proteasome or lysosome pathway.^118^ The degradation of p62 is controlled by autophagy at normal conditions, whereas oxidative stress activates p62 by the stimulation of p62-Keap1-Nrf2-ARE pathway.^119^ Like Nrf2 signaling pathway, the released Nrf2 induces the expression of some autophagy genes such as genes of autophagy initiation (ULK1), substrate recognition (SQSTM1 and CALCOCO2), autophagosome forming (ATG4D, ATG7, and GABARAPL1), autophagosome extension (ATG2B and ATG5), lysosomal clearance (ATG4D), suggesting Nrf2 can stimulate the autophagy.^120,121^ In addition, p62 initiates tau protein degradation by selective autophagy,^122^ and protect the neuronal homeostasis^123^ . Hence, the p62-Keap1-Nrf2 positive feedback axis can act as a neuroprotective mechanism^124^ and it connects the link between Nrf2 and autophagy pathways in AD pathogenesis^107^ (Figure 4).

### Crosstalk between autophagy and Nrf2 pathway: role of the gut microbiota

Recent evidence has demonstrated that modulation of the gut microbiota induces the potential beneficial effects on neurochemical pathways which can slow down AD progression.^125,126^ Bonfili et al. observed that four months of treatment in 8-week-old male 3×-Tg AD mice, the probiotic mixture (SLAB51) containing nine live bacterial strains (*Streptococcus thermophilus*, *Bifidobacteria* (*B. longum, B. breve, B. infantis*), *Lactobacilli* (*L. acidophilus, L. plantarum, L. paracasei, L. delbrueckii subsp. bulgaricus, L. brevis*) have neuroprotective effect on the initial stage of AD. In the treatment with SLAB51 in AD mice, autophagic markers such as beclin-1 and LC3-II were increased and the level of p62 was decreased, indicating the autophagic flux activation. SLAB51 treatment ameliorated brain damage with improved cognition and reduced levels of Aβ through the fractional recovery of ubiquitin-proteasome system and autophagy pathways.^125^ Te´gla´s et al. also found that using probiotic supplementation (with *Bifidobacterium longum* and *Lactobacillus acidophilus*) for twenty weeks, delayed the progression of AD in 3-month old, male APP/PS1 transgenic mice. They found that Nrf2 levels were elevated in the probiotic supplemented mice. Probiotics were found to stimulate the antioxidants such as superoxide dismutase (SOD)^126^ and finally it promotes cellular defense mechanism against oxidative stress in AD. Through the microbiota-gut-brain axis *Lactobacillus reuteri* can promote the production and absorption of indole-3-aldehyde and indole-3-propionic acid in to the brain^127^ that results in suppressed neuroinflammation and improved astrocyte activation. (Figure 4). Overall, it is clear that intervention with probiotics has a promising effect on suppressing oxidative stress and improving cognition in AD.

## Neuroprotection: role of the gut metabolites

The rising line of evidence expose that the gut microbiota and its metabolites such as polyphenols, SCFAs, antioxidants, vitamins etc. regulate many biosynthetic pathways which may have favorable or unfavorable effects on the host system (Table 2).^12^ Gut microbiota can change neurotransmitter function of Brain-Derived Neurotrophic Factor (BDNF) by either kynurenine pathway or the action and availability of SCFAs in the brain.^144^ Hence, potent gut microbiota is of utmost importance and can maintain neuronal health through antioxidative or anti-inflammatory pathways. The gut microbiome also regulates the penetrability of metabolites to BBB and improves intestinal barrier integrity; a hindrance to the intestinal gut microbiota colonization with pathogens, can reverse these protective effects.

**Table 2.** Common gut microbiota and their metabolites which act as neuroprotection in AD.

Name of Microbial derived metabolites	Name of Gut microbiota	References
Gamma-aminobutyric acid	*Lactobacillus brevis, Bifidobacterium dentium*	^128^
	*Bifidobacterium dentium*	^129,130^
	*Lactobacillus paracasei PF6, Lactobacillus delbrueckii subsp. bulgaricus PR1, Lactococcus lactis PU1, Lactobacillus plantarum C48, and Lactobacillus brevis PM17*	^130^
Serotonin	*Escherichia coli K-12*	^131^
Dopamine	*Escherichia coli K-12*	^131^
	*Prevotella, Bacteroides, Lactobacillus, Bifidobacterium,Clostridium,Enterococcus, Ruminococcus*	^132^
Norepinephrine,	*Escherichia coli K-12*	^131^
Acetylcholine	*Lactobacillus plantarum*	^133^
	*Lactobacillus plantarum, Bacillus subtilis, Escherichia coli, Staphylococcus aureus*	^134^
Short-chain fatty acids	*Bacteroides, Bifidobacterium, Propionibacterium*, *Eubacterium, Lactobacillus, Clostridium*, *Roseburia, Prevotella*	^135^
Histamine	*Morganella morganii, Klebsiella pneumoniae, Hafnia alvei*	^136,137^
	*Pediococcus parvulus, Lactobacillus mali Leuconostoc mesenteroides*	^137,138^
Vit K	*Escherichia coli, Klebsiella pneumoniae, Propionibacterium, Eubacterium*	^139^
Vit B2	*Bacillus subtilis, Escherichia coli*	^139^
Vit B9	*Bifidobacterium, Lactococcus lactis, Streptococcus thermophilus*	^139^
Vit B12	*Lactobacillus reuteri, Propionibacterium freudenreichi*	
Conjugated fatty acids	*Bifidobacterium, Butyrivibrio,Enterobacter, Lactobacillus, Clostridium, Citrobacter, Roseburia, Klebsiella, Megasphaera*	^140–142^
Indole derivatives	*Clostridium sporogenes, Escherichia coli,*	^143^

Recent studies revealed that Vit B and Vit K can improve neuronal health in the brain development and function.^139,145^ Deficiency in Vit B and Vit K correlated with decline in the memory functions of AD patients. Vit K2 (menaquinone-4) have antioxidative properties and markedly inhibits the rotenone-induced p38 activation, ROS production, and caspase-1 activity and finally reestablished mitochondrial membrane potential.^146^ Vit K is produced by *Escherichia coli*, *Klebsiella pneumoniae*, *Propionibacterium*, and *Eubacterium*; *Bacillus subtilis* and *E. coli* produces B2 (riboflavin); *Bifidobacterium, Lactococcus lactis*, and *Streptococcus thermophilus* produces B9 (folic acid); *Lactobacillus reuteri* and *Propionibacterium freudenreichii* produces B12 (cobalamin).^139^ There might be a great benefit in exploring these bacterial interaction with the host in the context of AD.

With the help of the gut bacteria, dietary amino acids (e.g. tyrosine, tryptophan, and phenylalanine) are metabolized into SCFAs, indole derivatives, neurotransmitters, organic acids, amines, and ammonia.^147^ The products of tryptophan metabolism such as tryptamine, tryptophan derivatives act as neuroactive molecule.^148^ Indole propanoic acid (an indole derivative) acts as antioxidant by beneficially influencing decline in neuroinflammation.^149^ Metabolized products of arginine and agmatine, ameliorates ROS production and indicates the therapeutic effects in CNS disorders.^150^ *In-vitro* and *in-vivo* studies also showed that agmatine defends astrocytes and microglia from the detrimental effect of oxidative stress.^151^ Useful metabolites like SCFAs formed by intestinal bacteria support to decrease ROS by controlling the activity of mitochondria.^12^ SCFAs decrease oxidative stress by declining microglial activation in the brain.^152^ In addition, SCFAs prevent neurotoxic Aβ aggregation in AD by obstracting Aβ40/42 assembly.^153^

The abundances of *Bifidobacterium spp*. decreases with increase in *Proteobacteria spp*. with growing age that might contribute to AD pathogenesis. *Bifidobacterium* have an important role in maintaining hippocampal plasticity and memory functions through the regulation of cholesterol levels by accumulating the serum leptin levels.^154,155^ Taken together, metabolites produced by the gut microbiota prevent and ameliorate oxidative stress associated with CNS depending upon the gut health of an individual.

Metabolic and neuroactive metabolites produced by gut microbiota have beneficial effects on the host health. Gut bacteria like *Lactobacilli* and *Bifidobacterium* can produce neurotransmitter such as γ-Aminobutyric acid (GABA) which can control glucose homeostasis and change the behavioral activity in the host.^156^ Tiwari V. et al. showed that synaptic plasticity in the hippocampus was changed by the reduced level of GABA with augmentation of glutamatergic neurotransmission in the AβPPswe-PS1dE9 mice model of AD.^157^ GABA-producing bacteria like *Lactobacilli* may recover the metabolic and depressive-like behavioral abnormalities in mice.^158^ In addition, *Cyanobacteria* produces β-methylamino-L-alanine (BMAA), neurotoxins causing cognitive impairment.^159^ Therefore, gut microbiota plays the main role in modulating ROS production in the CNS. The role of gut microbiota in modulating the host oxidative stress both peripherally and centrally looks very promising and needs further attention with more evidences to treat AD patients before symptoms occur in the brain.

## Conclusion

There are enough evidences from our own research and other research works as highlighted in this review that the interrelationship of the gut microbiota and the brain has controlled to transformative advances in neuroscience research. Gut dysbiosis acts as an important player in modulating the microbiota-gut-brain axis that may contribute to increased inflammation and accelerate amyloid β aggregation by TLR associate signaling cascade upon AD onset. Gut microbiota can influence AD pathogenesis via increased neuroinflammation elevated oxidative stress, dysregulated neurotransmitters, reduced SCFAs, elevated TLRs and increased toxins. All these pathways are interlinked. In this review, we primarily explored the role of dysbiotic gut microbiota on triggering oxidative stress and inflammation. In addition, we have also focused on studies stating maintenance of eubiosis by inhibition of pro-inflammatory cytokines, increasing anti-inflammatory cytokines, preventing oxidative stress and inflammation, and improving bacterial metabolites with beneficial function to improve host health. To date, most of the gut microbiome research work mainly emphasize the connections between the gut microbiota and AD, although the contributory relationship between specific bacteria and the brain dysfunction in AD are not demonstrated functionally. Therefore, the specific function of the precise gut microbiota in AD patients persist as indefinable. If we could understand these interactions and molecular mechanisms in detail then the commensal gut bacteria can be utilized as targets for novel noninvasive diagnosis and future treatment strategies of AD. Furthermore, increasing the usage of germ-free, specific gene knockout and humanized sporadic AD animal models for the characterization of the gut microbiota and its cross-communication with the host in AD pathophysiology is needed. Targeting the potential beneficial bacteria (as described in this review) as a novel intervention strategy can prohibit or slow AD onset or counteract its development. In addition, new treatment strategies such as fecal microbiota transfer, probiotics (beneficial bacterial cocktails), beneficial metabolite supplementations to improve the brain and the gut health in pre-symptomatic AD patients may have translational value. Therefore, efficacy of the gut microbiota and its effective metabolites in neurodegenerative and neuroprotective mechanisms is continued to be open for further investigation. Therefore, it may provide new suggestions in the pathology and treatment to AD.
